# Homology-feature-assisted quantification of fibrotic lesions in computed tomography images: a proof of concept for CT image feature-based prediction for gene-expression-distribution

**DOI:** 10.1007/s11548-025-03428-8

**Published:** 2025-05-28

**Authors:** Kentaro Doi, Hodaka Numasaki, Yusuke Anetai, Yayoi Natsume-Kitatani

**Affiliations:** 1 National Institutes of Biomedical Innovation, Health and Nutrition, Osaka, Japan; 2 Graduate School of Medicine, The University of Osaka, Osaka, Japan; 3 Department of Radiology, Kansai Medical University, Osaka, Japan; 4 Institute of Advanced Medical Sciences, Tokushima University, Tokushima, Japan; 5 Institute for Protein Research, The University of Osaka, Osaka, Japan

**Keywords:** Idiopathic interstitial pneumonias, Quantitative image analysis, Homology, CT image, Computer-aided diagnosis

## Abstract

**Purpose:**

Computed tomography (CT) image is promising for diagnosing of interstitial idiopathic pneumonias (IIPs); however, quantification of IIPs lesions in CT images is required. This study aimed to quantitatively evaluate fibrotic lesions in CT images using homology-based image analysis.

**Methods:**

We collected publicly available CT images comprising 47 fibrotic images and 36 non-fibrotic images. The homology-profile (HP) image analysis method provides b0 and b1 profiles, indicating the number of isolated components and holes in a binary image. We locally applied the HP method to the CT image and generated homology-based feature (HF) maps as resultant images. The collected images were randomly divided into the tuning dataset and the testing dataset. The cut-off value for classifying the HF map for fibrotic or non-fibrotic images was defined using receiver operating characteristic (ROC) analysis with the tuning dataset. This cut-off value was evaluated using the testing dataset with accuracy, sensitivity, specificity, and precision.

**Results:**

We successfully visualized the quantification of fibrotic lesions in the HF map. The b0 HF map was more suitable for quantifying fibrotic lesions than b1. The mean cut-off value of the b0 HF map was 199, with all performances achieved at 1.0. Furthermore, the classification of the b0 HF map for fibrotic or lung cancer images achieved all maximum performances at 1.0.

**Conclusion:**

This study demonstrated the feasibility of using the HF in quantitatively evaluating fibrotic lesions in CT images. Our proposed HP-based method can also be promising in quantifying the fibrotic lesions of patients with IIPs, which can be applicable to assist the diagnosis of IIPs.

**Supplementary Information:**

The online version contains supplementary material available at 10.1007/s11548-025-03428-8.

## Introduction

Idiopathic interstitial pneumonia (IIP) is a fatal disease with no clear cause known and no cure. There are various categories, namely, mainly idiopathic pulmonary fibrosis (IPF), idiopathic nonspecific interstitial pneumonia, cryptogenic organizing pneumonia, respiratory bronchiolitis-associated interstitial lung disease (RB-ILD), desquamative interstitial pneumonia, and acute interstitial pneumonia [1]. As for the treatment, there is a large difference of strategies, and the prognosis depends on the category. The prognosis of the patients with IPF is significantly poorer than that with non-IPF IIPs. In addition, the acute exacerbation of IPF is associated with a high mortality rate [2, 3].

High-resolution computed tomography (HRCT) images are commonly used to diagnose IIPs definitively, and the HRCT image provides the typical findings (e.g., extensive traction bronchiectasis or honeycomb lung) which indicate the IPF [4–7]. Those typical findings can be able to aid diagnosis in the HRCT image; however, it is challenging to classify the category from the HRCT image accurately even though the radiological experts exert their skill. Moreover, an inter-observer variability can lead to diagnostic limitations [4]. Therefore, a valid quantification method for fibrosis findings in the HRCT image could be a useful to assist the deciding the category and diagnosis for IIPs.

**Fig. 1:**
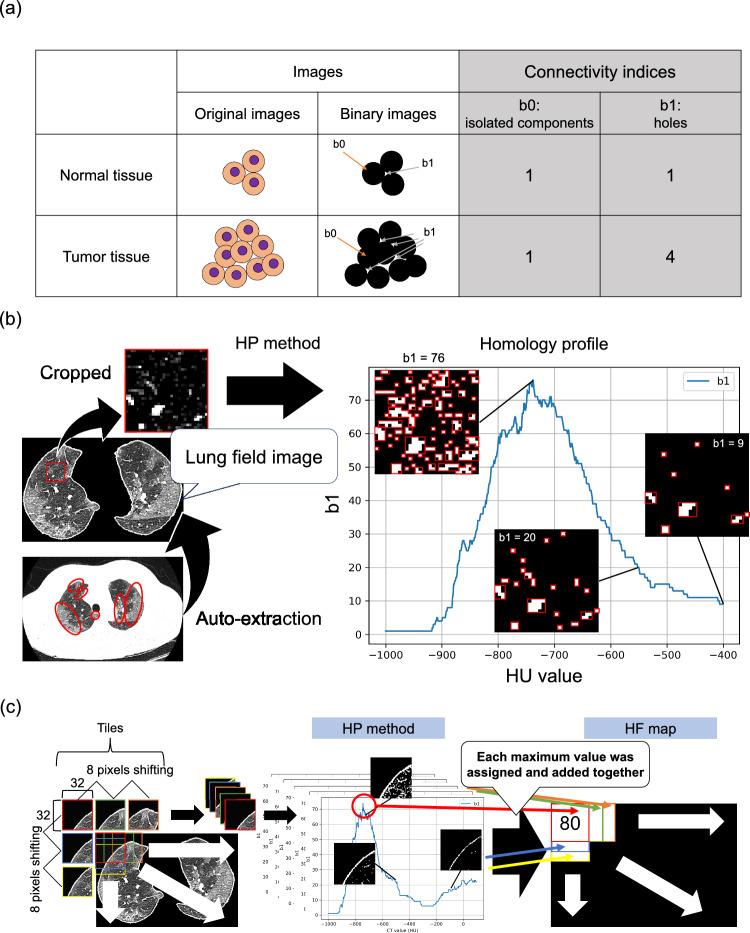
A concept of the HP method in this study. **a** counting Betti numbers composed of b0 and b1 in a binary image processed with a threshold value. **b** the extracted lung field image and the HP method that continuously counts the Betti numbers in binary images by changing the threshold value along with the HU value. Red circles indicate fibrotic lesions. The red rectangle in the lung field image is an example of a tile cropped for analysis using the HP method. Each red rectangle in the binary images indicates each hole, and the number of the holes is annotated. **c** the procedure of tile-shifted HP method. Abbreviations: HP, homology-profile; CT, computed tomography; HU, Hounsfield unit; and HF, homology-based feature

Homology-based image analysis has been reported to be effective to detect the certain features of medical images such as pathological and radiological images [8–10]. Nakane et al. proposed a homology-profile (HP) method to detect the tumor cell in the hematoxylin–eosin-stained pathological image [8]. It has been demonstrated that the homology-based feature (HF) is effective in capturing the CT imaging feature and can improve the performance of radiomic analyses [9]. Betti numbers composed of b0 and b1 are obtained by using the HP method, namely, HFs. These Betti numbers indicate the number of isolated components and the number of holes in a binary image, respectively (Fig. 1a). This calculation is performed by changing the threshold value applied to the image binarization. As a result of the calculation, the connectivity of objects in an image is quantified. Ninomiya and Arimura showed that the HFs from CT images are an effective feature in the prognostic prediction of lung cancer patients [10]. They demonstrated that the HFs are helpful in extracting the topologically invariant morphological features of lung cancers in CT images, which could be included in patterns of tumor inhomogeneity. Therefore, the HFs may extract the practical features in quantifying fibrotic lesions in CT images from the pattern of fibrosis homogeneity.

Hereby, we have hypothesized that the HFs may be promising in quantification of the fibrotic lesion of IIPs in the HRCT image. CT images of the COVID-19 case also display fibrotic lesions as a typical finding which is similar to fibrotic lesions of IIPs. This study shows a proof of concept for quantifying the fibrotic lesion of IIPs in the HRCT image by applying the HP method to CT images of the COVID-19 cases.

## Materials and methods

### Data collecting

**Fig. 2:**
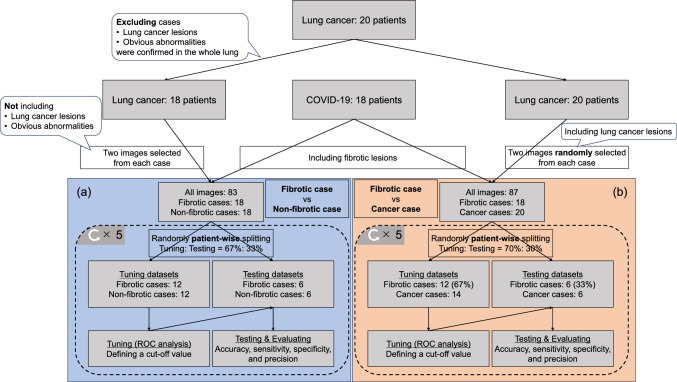
A scheme of data collecting and a concept for this study. **a** and **b** indicate the classification between fibrotic cases and non-fibrotic cases and fibrotic cases and lung cancer cases, respectively. Abbreviations: ROC, receiver operative characteristics

Eighteen CT image datasets of patients with COVID-19 and 20 radiotherapy datasets for lung cancer consisting of CT images and radiotherapy structures were obtained from the Cancer Image Archive [11, 12]. We selected CT datasets of COVID-19 according to the following criteria: the patients underwent a non-contrast-enhanced CT examination; the manufacturer of the CT equipment was GE Health Care LTD.; and the CT images did not display pleural fluid in the lung field. From the COVID-19 cases, we selected forty-seven CT images that show obvious fibrotic lesions and used them for classification in this study. The association between the cause of lung cancer and interstitial lung abnormalities (ILAs), such as ground-glass opacity, has been reported, the ILAs can be observed in the CT images of patients with lung cancer [13]. These ILAs lesions can be a noise for quantitatively evaluating the fibrotic lesions. As lung cancer lesions and/or obvious lung abnormalities were observed in the whole lung of two patients with lung cancer, these cases were excluded when classifying fibrotic and non-fibrotic cases. The excluded cases were used to classify fibrotic and lung cancer cases. A total of thirty-six CT images were acquired by selecting two CT images from each lung cancer case, these images did not include lung cancer lesions defined by radiologists and/or any lung abnormalities. The data collection scheme is illustrated in Fig. 2. The patient demographic data are presented in Table 1.

**Table 1:** Patient demographic data

Characteristic	COVID-19 (n = 18)	Lung cancer (n = 20)	Overall (n = 38)
Age (median [25^th^–75th percentile])	60 (46.5–69)	NA	NA
Sex (male/female)	10/8	12/8	22/16
Fibrosis vs non-fibrosis	10/8	10^*^/8	20/16
Fibrosis vs lung cancer	10/8	12/8	22/16

The age of patients with lung cancer was not described in this dataset. *Two cases were excluded because lung cancer lesions and/or obvious abnormalities were confirmed in the whole lung

Abbreviation: NA, not applicable

### Preprocessing

These collected CT images underwent the following preprocessing. First, the mask image covering the lung field was created from the CT image with *numpy*, *scipy,* and *scikit-image*. Second, the lung field image was extracted by using the mask image, as shown in Fig. 1b.

### Obtaining HF map

The HFs are composed of b0 and b1 indicating the connectivity of isolated components and connectivity of holes in a binary image, as shown in Fig. 1a. These indices are calculated by using the HP method, in which changing the threshold value is applied to the image binarization (Fig. 1b). The HP calculation was executed with the in-house HP software. The calculation region of interest was set to a 32 × 32 matrix, named a tile. The tile was shifted every eight pixels on the CT image through the entire 512 × 512 matrix, and the maximum value of HP derived from each tile was assigned to corresponded region in the output image. Here, the matrix size of tile and the shifting size were validated (Online Resource 1), and they have been decided. When pixels on the calculation tiles were overlapped, the assigned maximum values were summed. This output image is called HF map in this study. Each HP was standardized by dividing it by its own pixel size. The range of threshold values was set from − 700 to − 400 Hounsfield unit (HU) because the range has been reported to be a representative range of the fibrotic lesion of IPF [14]. When the ratio of pixels contained in the calculation tile was over 95%, the calculation was systematically skipped. This tile-shifted HP method is detailed in Fig. 1c.

### Cut-off value for classification of fibrosis or non-fibrosis

We conducted slice-wise classification of a CT image as a fibrosis or non-fibrosis based on the maximum value of b0 HF map. First, the collected dataset was randomly patient-wise split into two datasets for tuning the cut-off value (12/18: 67%) and testing (6/18: 33%), as shown in Fig. 2a. Second, the cut-off value was defined with the slice-wise receiver operating characteristic (ROC) analysis by using tuning dataset and Youden Index. Third, the cut-off value was slice-wise tested and evaluated using the testing dataset for the accuracy, sensitivity, specificity, and precision score. The above procedures were repeated 5 times, and these cut-off value and these performances were averaged. In addition, the slice-wise classification of a CT image as fibrosis or lung cancer was conducted using the same procedure as the above. Two excluded cases in the former classification were also included in the latter classification, as shown in Fig. 2b. It validated the feasibility of the b0 HF map in quantitatively classifying even if CT images including lung cancer lesions and those abnormalities.

### Development environment

This methodology was implemented by Python version 3.11.7, *numpy* version 1.26.4, *pydicom* version 2.4.4, *scipy* version 1.11.4, *skimage* 0.22.0, and *opencv* version 4.9.0. This calculation was executed on the CPU of 13th Gen Intel (R) Core (TM) i9-13900H 2.6 GHz and the super-computing resource provided by Human Genome Center, the Univ. of Tokyo (http://sc.hgc.jp/shirokane.html).

## Results

### HF map

**Fig. 3:**
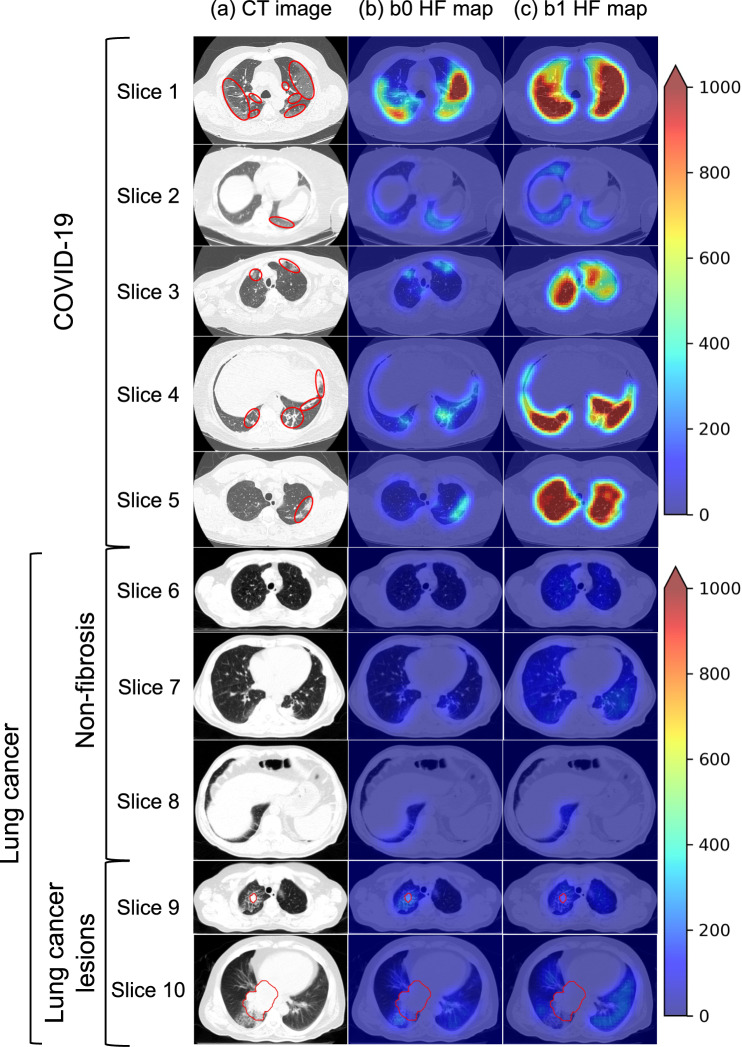
Examples of the HF maps. **a**, **b** and **c** show the CT image, b0 HF map, and b1 HF map, respectively. The area annotated with the red line in the CT images indicates fibrotic lesions. The colorbar presents the absolute HF map value. Here, the HF map value is adjusted from 0 to 1000 to compare the b0 HF and b1 HF maps with the same scale. The HF maps show that b0 is superior to b1 in quantifying fibrotic lesions in the CT images. Red line indicates lung cancer lesions defined by radiologists. Abbreviations: CT, computed tomography; HF, homology-based feature

HF maps were acquired by the tile-shifted HP method, as shown in Fig. 3. According to the HF maps (Fig. 3), the b0 HF has the highest association with the fibrotic lesions. The b0 HF map more specifically detected fibrotic lesions than the b1 HF maps, and the value covering the fibrotic lesion depends on the density of the fibrosis, the fibrotic type, or surrounding lung nodules (e.g., vessels). In contrast, almost whole lung field was enhanced even though the area included the nodules in the b1 HF map. Thus, we validated the conditions for obtaining HF maps that can detect fibrotic lesions in CT images and qualitatively confirmed that b0 could clearly detect fibrotic lesions more than b1. Furthermore, we also validated a method that applied the argmax value, but not the maximum value, of the HP; however, we confirmed that it was not suitable for detecting fibrotic lesions (Online Resource 2). For these reasons, we decided to use b0 to generate HF maps in this study.

### Cut-off value for slice-wise classification of fibrosis or non-fibrosis

**Fig. 4:**
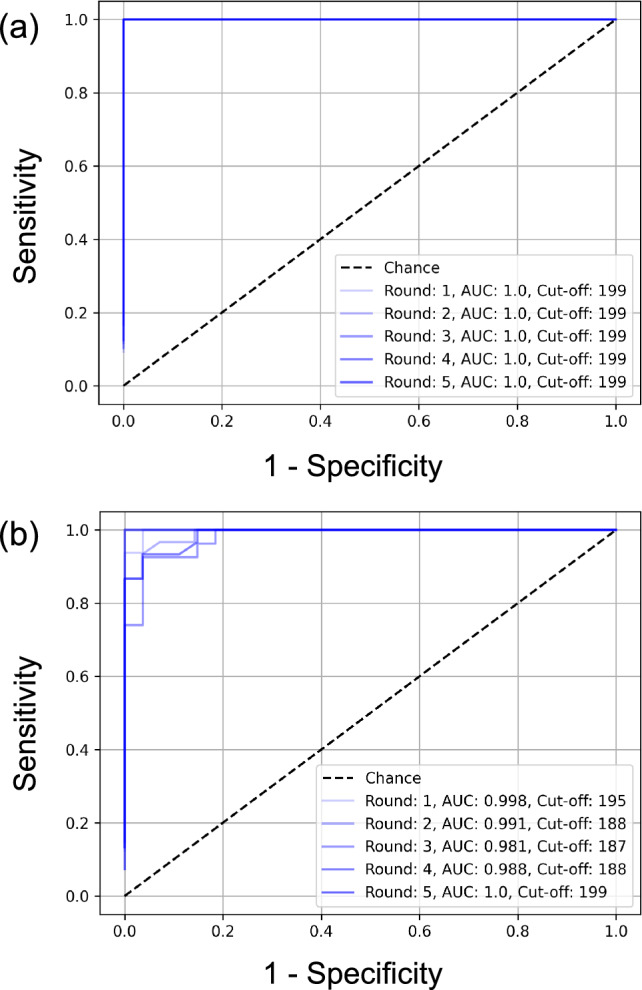
ROC curves for defining the cut-off value. **a** the ROC curves for classifying fibrotic and non-fibrotic cases. **b** the ROC curves for classifying fibrotic and lung cancer cases. Abbreviations: ROC, receiver operative characteristics; AUC, area under the ROC curve

According to the ROC analysis using the tuning datasets, the mean ± standard deviation (SD) of the cut-off value and area under the ROC curve (AUROC) were 199 ± 0 and 1.00 ± 0, respectively. Figure 4a depicts the ROC curves. As a result of the slice-wise classification using the testing datasets based on a cut-off value in each experiment, the mean ± SD of accuracy, sensitivity, specificity, and precision were 1.00 ± 0, 1.00 ± 0, 1.00 ± 0, and 1.00 ± 0, respectively. These results have shown the feasibility of b0 HF map applying to classify the CT image for fibrosis or non-fibrosis.

### Cut-off value for slice-wise classification of fibrosis or lung cancer

The mean ± SD of the cut-off value and AUROC were 191.4 ± 5.32 and 0.992 ± 0.008, respectively, based on the result of the ROC analysis for the tuning dataset (Fig. 4b). Thus, there were no substantial differences in the b0 HF map cut-off values used to classify fibrotic or non-fibrotic lung lesions and those used to differentiate lung cancer from fibrotic lesions. According to the slice-wise classification using the testing dataset, the mean ± SD of accuracy, sensitivity, specificity, and precision were 0.892 ± 0.059, 1.00 ± 0, 0.721 ± 0.158, and 0.854 ± 0.068, respectively. These maximum performances have achieved an accuracy of 0.966, sensitivity of 1.00, specificity of 0.917, and precision of 0.944 (Table 2). These findings indicate that the b0 HF map value could also be applied to discriminate between fibrotic and lung cancer lesions.

**Table 2:** Comparison between previous studies and our proposed methodology

Ref. No	Accuracy (%)	Sensitivity (%)	Specificity (%)	Precision (%)
24	99.5	100	99.0	NA
25	98.9	99.0	98.9	98.9
26	99.4	99.6	99.2	NA
Ours (vs non-fibrosis)	100	100	100	100
Ours (vs lung cancer)	96.6	100	91.7	94.4

Abbreviation: Ref., reference; NA, not applicable

## Discussion

We have successfully quantified the fibrotic lesion of the COVID-19 case displayed on the CT image by using the HF in this study. The association between the HF and the fibrotic lesion in the CT image has been demonstrated. The HFs are composed of the b0 and b1, which equal the number of isolated components (black pixels) and the number of holes (white pixels) in a binary image. These indices represent the connectivity of object in an image. Our developed b0 HF map visualizes the fibrotic lesion with a higher value than that of the non-fibrotic lung field and lung cancer nodule. This demonstrated that the feasibility of the b0 HF map value in classifying CT images into fibrosis or non-fibrosis and lung cancer case. Hence, we found that b0 HF, the connective caused by the number of isolated components, could capture the topological features that lead to the quantification of inhomogeneity differences between fibrotic and non-fibrotic lung fields. On the other hand, our proposed method misclassified the lung cancer cases (slices 9 and 10 in Fig. 3) complicated with fibrotic lesions. It has probably been caused by the ILAs related to lung cancer, as Hata et al. mentioned [13], and the similarity of the fibrotic lesions and the ILAs has been shown based on the HF map value in this study. To address the complicated case, the ILA lesions may be discriminated from lung cancer lesions by pixel-wise applying the cut-off value to the b0 HF map. Thus, this study has demonstrated the feasibility of homology-based image analysis in quantitatively evaluating fibrotic lesions in the CT image.

There have been many studies that can quantitatively evaluate the fibrotic lesions in the CT images. These methods have been based on histogram features and/or texture features of the CT image and have been frequently applied to many studies to quantify the fibrotic lesion in the CT images [15–19]. While the methods are promising in quantifying the fibrotic lesion, those performances are influenced by CT imaging conditions (e.g., KVp and slice thickness) [20]. It may be more challenging to correctly regulate the histogram-based features than that of the HF since the feature of the histogram is simply derived from the histogram of CT images. These CT imaging conditions could also influence the performance of our proposed method. However, the HP method can quantify the connectivity of objects in the image, and the effect can be flexibly reduced by optimizing the calculation parameters (e.g., the range of the HP calculation). Therefore, our proposed methodology is probably more robust than the past reported studies based on the histogram and/or texture analysis. Moreover, machine learning (ML)-based methods have also been proposed [4, 21–26]. Regarding the classification of CT images as fibrotic or non-fibrotic, previous supervised ML-based studies have shown an accuracy of approximately 0.99 [24–26]. Thus, ML-based techniques show excellent performances, but the performance using supervised ML depends on the quantity of training datasets. Our proposed methodology provides the excellent performances comparable to these ML-based studies, as shown in Table 2; however, a larger cohort size is required to accurately compare the performances in this study. While ML-based classification and segmentation for fibrotic lesions in the CT image are less challenging than other strategies, it may be challenging to develop the absolute quantification of the fibrotic lesions with only CT image features, like the HF map. Our proposed method is thought to reflect the actual types, densities, and morphologies of cells, so it is possible that they can be used to find fibrotic lesions without relying on prior knowledge, along with values (b0) that indicate their characteristics. Thus, it has been shown that it is possible to create features based on cell biology and mathematics, even without a large number of images or annotations by experts.

The current study has a few limitations. First, our proposed method is influenced by the pixel size of the CT image while a standardization based on the pixel size was implemented to overcome it in this study. In the future, this standardization method will be completely developed. However, the standardization must not be considered if the analysis is specified to IIPs in the HRCT image. Second, the analysis resolution of the proposed method is not high. This issue happens due to setting the shift parameter in the tile-shifted HP calculation to 8 pixels. If the shift size of the tile is smaller, a higher resolution analysis result can be obtained. Although the high-resolution result is acquired due to the optimization of this calculation, then time consumption can also be an issue, which our future study can dissolve. Third, these cases are COVID-19, which we used in this study, but not the cases of IIPs. There has a difference in detailed characteristics of the fibrotic lesion between the COVID-19 case and IIPs, so we must validate the feasibility of this methodology in quantitative evaluation for the fibrotic lesions in IIPs patient in the near future.

CALIPER (Computer-Aided Lung Informatics for Pathology Evaluation and Rating) is the representative model that quantifies fibrotic lesions in the CT image. This model visualizes typical findings in the HRCT image (e.g., reticular or honeycombing) by using each color [19]. While CALIPER is considered one of the state-of-the-art models for quantifying fibrotic lesions in CT images, CALIPER quantifies fibrotic lesions based on the histogram information of CT images. The histogram information is directly affected by imaging conditions; however, HF maps are assumed to be less affected because the HF map is based on homological features that are not directly related to imaging conditions. Thereby, it is possible that the quantification by the HF map is superior to CALIPER in terms of quantifying fibrotic lesions in CT images. Although the HF map is thought to be a promising method for quantitatively evaluating fibrotic lesions in CT images, the HF map value also needs to clarify the correlation with the typical HRCT image findings and clinical examination values (e.g., forced vital capacity), like CALIPER. It is expected that diffusion equation quantification (DEQ) model [27] could also improve our proposed HF maps with contrast enhancement in lung lesions. In the future, we will improve the proposed HF map by using the DEQ model and HRCT images of IIPs, which are pathologically confirmed, to quantify fibrotic lesions of each IIP type and typical findings in HRCT images. Furthermore, we eventually aim to visualize the gene expression information relating to IIPs on the HRCT image by quantitatively associating the HF map value and the local gene expression information obtained by using the bulk RNA-seq analysis.

## Conclusions

This study has demonstrated the feasibility of the homology-based feature in the quantitative evaluation of the fibrotic lesions in the CT image. This quantification of the fibrotic lesion may be promising in establishing a quantitative relationship between the HF map value and the local gene expression in the CT image. It can assist in diagnostic imaging for fibrotic lesions without extra examination and reduce the burden on patients and clinicians. We will validate the feasibility of the HF map in quantitatively evaluating the fibrotic lesion of IIPs cases that have already been collected in our institute.

## Supplementary Information

Below is the link to the electronic supplementary material.

Supplementary file1 (PDF 344 KB)

Supplementary file2 (PDF 199 KB)

## References

[1] Travis WD, Costable U, Hansell DM et al (2013) An official American Thoracic Society/European Respiratory Society statement: update of the international multidisciplinary classification of the idiopathic interstitial pneumonias. Am J Respir Crit Care Med 188(6):733–748. 10.1164/rccm.201308-1483ST 24032382 10.1164/rccm.201308-1483ST PMC5803655

[2] Kato M, Yamada T, Kataoka S, Arai Y, Miura K, Ochi Y, Ihara H, Koyama R, Sasaki S, Takahashi K (2019) Prognostic differences among patients with idiopathic interstitial pneumonias with acute exacerbation of varying pathogenesis: a retrospective study. Respir Res 20:1–14. 10.1186/s12931-019-1247-z 31852459 10.1186/s12931-019-1247-z PMC6921398

[3] Song JW, Hong SB, Lim CM, Koh Y, Kim DS (2011) Acute exacerbation of idiopathic pulmonary fibrosis: incidence, risk factors and outcome. Eur Respir J 37(2):356–363. 10.1183/09031936.00159709 20595144 10.1183/09031936.00159709

[4] Koh SY, Lee JH, Park H, Goo JM (2024) Value of CT quantification in progressive fibrosing interstitial lung disease: a deep learning approach. Eur Radiol 34(7):4195–4205. 10.1007/s00330-023-10483-9 38085286 10.1007/s00330-023-10483-9

[5] George PM, Spagnolo P, Kreuter M, Altinisik G, Bonifazi M, Martinez FJ, Molyneaux PL, Renzoni EA, Richeldi L, Tomassetti S, Valenzuela C, Vancheri C, Varone F, Cottin V, Costable U (2020) Progressive fibrosing interstitial lung disease: clinical uncertainties, consensus recommendations, and research priorities. Lancet Respir Med 8(9):925–934. 10.1016/S2213-2600(20)30355-6 32890499 10.1016/S2213-2600(20)30355-6

[6] Wijsenbeek M, Kreuter M, Olson A, Fischer A, Bendstrup E, Wells CD, Denton CP, Mounir B, Zouad-Lejour L, Quaresma M, Cottin V (2019) Progressive fibrosing interstitial lung diseases: current practice in diagnosis and management. Curr Med Res Opin 35(11):2015–2024. 10.1080/03007995.2019.1647040 31328965 10.1080/03007995.2019.1647040

[7] Walsh SL, Sverzellati N, Devaraj A, Wells AU, Hansell DM (2012) Chronic hypersensitivity pneumonitis: high resolution computed tomography patterns and pulmonary function indices as prognostic determinants. Eur Radiol 22:1672–1679. 10.1007/s00330-012-2427-0 22466512 10.1007/s00330-012-2427-0

[8] Nakane K, Takiyama A, Mori S, Matsuura N (2015) Homology-based method for detecting regions of interest in colonic digital images. Diagn Pathol 10:1–5. 10.1186/s13000-015-0244-x 25907563 10.1186/s13000-015-0244-x PMC4448533

[9] Kadoya N, Tanaka S, Kajikawa T, Tanabe S, Abe K, Nakajima Y, Yamamoto T, Takahashi N, Takeda K, Dobashi S, Takeda K, Nakane K, Jingu K (2020) Homology-based radiomic features for prediction of the prognosis of lung cancer based on CT-based radiomics. Med Phys 47(5):2197–2205. 10.1002/mp.14104 32096876 10.1002/mp.14104

[10] Ninomiya K, Arimura H (2020) Homological radiomics analysis for prognostic prediction in lung cancer patients. Phys Med 69:90–100. 10.1016/j.ejmp.2019.11.026 31855844 10.1016/j.ejmp.2019.11.026

[11] Saltz J, Saltz M, Prasanna P, Moffitt R, Hajagos J, Bremer E, Balsamo J, Kurc T (2021) Stony brook university COVID-19 positive cases. Cancer Imaging Arch. 10.7937/TCIA.BBAG-2923

[12] Hugo GD, Weiss E, Sleeman WC, Balik S, Keall PJ, Lu J, Williamson JF (2016) Data from 4D lung imaging of NSCLC patients (Version 2). Cancer Imaging Arch. 10.7937/K9/TCIA.2016.ELN8YGLE

[13] Hata A, Hino T, Yanagawa M, Nishino M, Hida T, Hunninghake GM, Tomiyama N, Christiani DC, Hatabu H (2022) Interstitial lung abnormalities at CT: subtypes, clinical significance, and associations with lung cancer. Radiographics 42(7):1925–1939. 10.1148/rg.220073 36083805 10.1148/rg.220073 PMC9630713

[14] Capaccione KM, Wang A, Lee SM, Patel N, Austin JH, Maino P, Padilla M, Salvatore MM (2021) Quantifying normal lung in pulmonary fibrosis: CT analysis and correlation with% DLCO. Clin Imaging 77:287–290. 10.1016/j.clinimag.2021.06.021 34171742 10.1016/j.clinimag.2021.06.021

[15] Robbie H, Daccord C, Chua F, Devaraj A (2017) Evaluating disease severity in idiopathic pulmonary fibrosis. Eur Respir Rev 26:145. 10.1183/16000617.0051-2017 10.1183/16000617.0051-2017 PMC9488723 28877976

[16] Hartley PG, Galvin JR, Hunninghake GW, Merchant JA, Yagla SJ, Speakman SB, Schwartz DA (1994) High-resolution CT-derived measures of lung density are valid indexes of interstitial lung disease. J Appl Physiol 76(1):271–277. 10.1152/jappl.1994.76.1.271 8175517 10.1152/jappl.1994.76.1.271

[17] Best AC, Lynch AM, Bozic CM, Miller D, Grunwald GK, Lynch DA (2003) Quantitative CT indexes in idiopathic pulmonary fibrosis: relationship with physiologic impairment. Radiology 228(2):407–414. 10.1148/radiol.2282020274 12802000 10.1148/radiol.2282020274

[18] Sverzellati N, Calabrò E, Chetta A, Concari G, Larici AR, Mereu M, Cobelli R, De FM, Zompatori M (2007) Visual score and quantitative CT indices in pulmonary fibrosis: relationship with physiologic impairment. Radiol Med (Torino) 112(8):1160–1172. 10.1007/s11547-007-0213-x 18193399 10.1007/s11547-007-0213-x

[19] Maldonado F, Moua T, Rajagopalan S, Karwoski RA, Raghunath S, Decker PA, Hartman TE, Bartholmai BJ, Robb RA, Ryu JH (2013) Automated quantification of radiological patterns predicts survival in idiopathic pulmonary fibrosis. Eur Respir J 43(1):204–212. 10.1183/09031936.00071812 23563264 10.1183/09031936.00071812

[20] Wu X, Kim GH, Salisbury ML, Barber D et al (2019) Computed tomographic biomarkers in idiopathic pulmonary fibrosis. The future of quantitative analysis. Am J Respir Crit Care Med 199(1):12–21. 10.1164/rccm.201803-0444PP 29986154 10.1164/rccm.201803-0444PP

[21] Hansell DM, Goldin JG, King TE, Lynch DA, Richeldi L, Wells AU (2015) CT staging and monitoring of fibrotic interstitial lung diseases in clinical practice and treatment trials: a position paper from the Fleischner Society. Lancet Respir Med 3(6):483–496. 10.1016/S2213-2600(15)00096-X 25975761 10.1016/S2213-2600(15)00096-X

[22] Thillai M, Oldham JM, Ruggiero A, Kanavati F, McLellan T, Saini G, Johnson SR, Ble FX, Azim A, Ostridge K, Platt A, Belvisi M, Maher TM, Molyneaux PL (2024) Deep learning–based segmentation of computed tomography scans predicts disease progression and mortality in idiopathic pulmonary fibrosis. Am J Respir Crit Care Med 210(4):465–472. 10.1164/rccm.202311-2185OC 38452227 10.1164/rccm.202311-2185OC PMC11351794

[23] Shin B, Oh YJ, Kim J, Park SG, Lee KS, Lee HY (2024) Correlation between CT-based phenotypes and serum biomarker in interstitial lung diseases. BMC Pulm Med 24(1):523. 10.1186/s12890-024-03344-8 39427156 10.1186/s12890-024-03344-8 PMC11490112

[24] Pham TD (2020) A comprehensive study on classification of COVID-19 on computed tomography with pretrained convolutional neural networks. Sci Rep 10(1):16942. 10.1038/s41598-020-74164-z 33037291 10.1038/s41598-020-74164-z PMC7547710

[25] Gupta K, Bajaj V (2023) Deep learning models-based CT-scan image classification for automated screening of COVID-19. Biomed Signal Process Control 80:104268. 10.1016/j.bspc.2022.104268 36267466 10.1016/j.bspc.2022.104268 PMC9556167

[26] Salama GM, Mohamed A, Abd-Ellah MK (2024) COVID-19 classification based on a deep learning and machine learning fusion technique using chest CT images. Neural Comput Appl 36(10):5347–5365. 10.1007/s00521-023-09346-7

[27] Anetai Y, Doi K, Takegawa H, Koike Y, Yui M, Yoshida A, Hirota K, Yoshida K, Nishio T, Kotoku J, Nakamura M, Nakamura S (2024) Diffusion equation quantification: selective enhancement algorithm for bone metastasis lesions in CT images. Phys Med Biol 69(24):245007. 10.1088/1361-6560/ad965c 10.1088/1361-6560/ad965c 39577089

