# Supplementary material for: Homology-feature-assisted quantification of fibrotic lesions in computed tomography images: a proof of concept for CT image feature-based prediction for gene-expression-distribution
Source: Int J Comput Assist Radiol Surg. 2025 May 28;20(8):1703–11. doi: 10.1007/s11548-025-03428-8 (PMC12350597; doi:10.1007/s11548-025-03428-8)
Supplement: Supplementary file 1 — Supplementary file1 (PDF 344 KB) [file 11548_2025_3428_MOESM1_ESM.pdf]

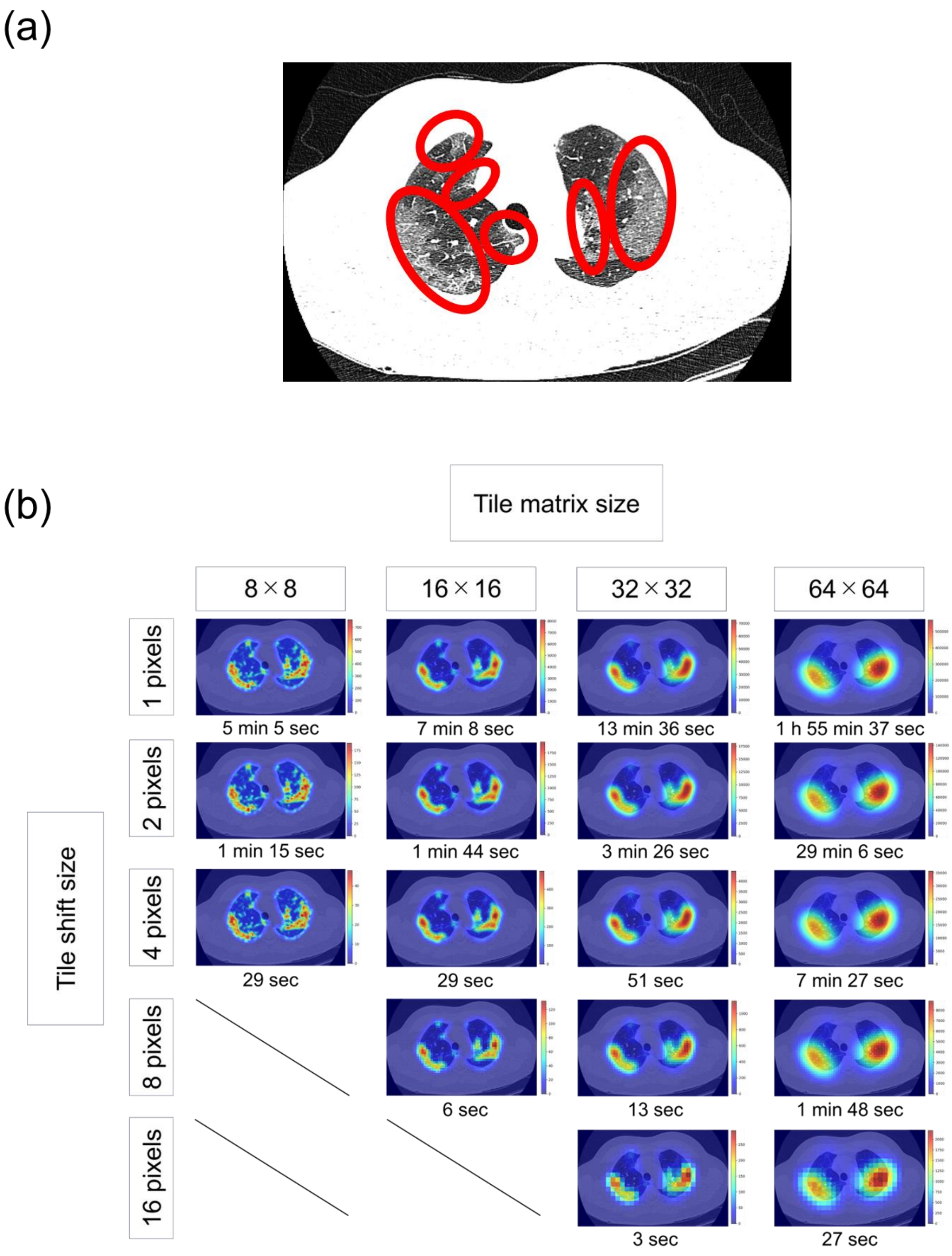

Online Resource 1. Results of a preliminary validation of calculation conditions of the tile-shifted HP method. (a) depicts an example CT image we used for the validation. Red circles indicate fibrotic lesions. (b) represents the results of the validation. We validated the tile matrix and tile-shift sizes for the tile-shifted HP method. Each calculation time for each condition is written below each resultant image. According to these results, the tile matrix size and the tile shift size have been decided as a 32×32 matrix and 8 pixels, respectively. Although the HF map calculated with the tile matrix size of 16×16 and the tile shift size of 8 pixels also demonstrated an excellent coverage performance on the fibrotic lesions and a quick calculation, the differences of the maximum value and minimum value were not large. That is why applying the latter condition was thought to be challenging to

discriminate depending on the quantitative fibrotic degree. If the calculation tile matrix was not overlapped in a validating condition (i.e., the tile shift size > one side of the tile matrix), the validation was skipped.

Abbreviations: HP, homology-profile; h, hour; min, minutes; and sec, seconds.
