# Supplementary material for: Homology-feature-assisted quantification of fibrotic lesions in computed tomography images: a proof of concept for CT image feature-based prediction for gene-expression-distribution
Source: Int J Comput Assist Radiol Surg. 2025 May 28;20(8):1703–11. doi: 10.1007/s11548-025-03428-8 (PMC12350597; doi:10.1007/s11548-025-03428-8)
Supplement: Supplementary file 2 — Supplementary file2 (PDF 199 KB) [file 11548_2025_3428_MOESM2_ESM.pdf]

(a)

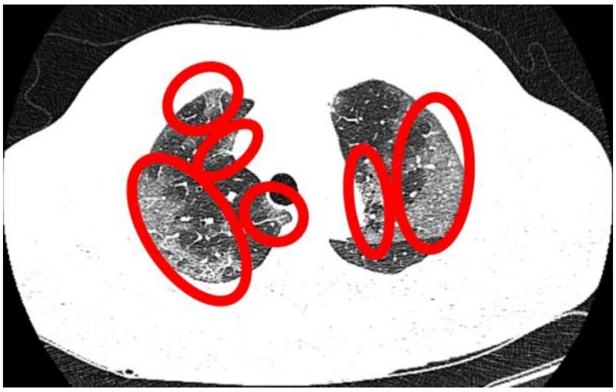

(b)

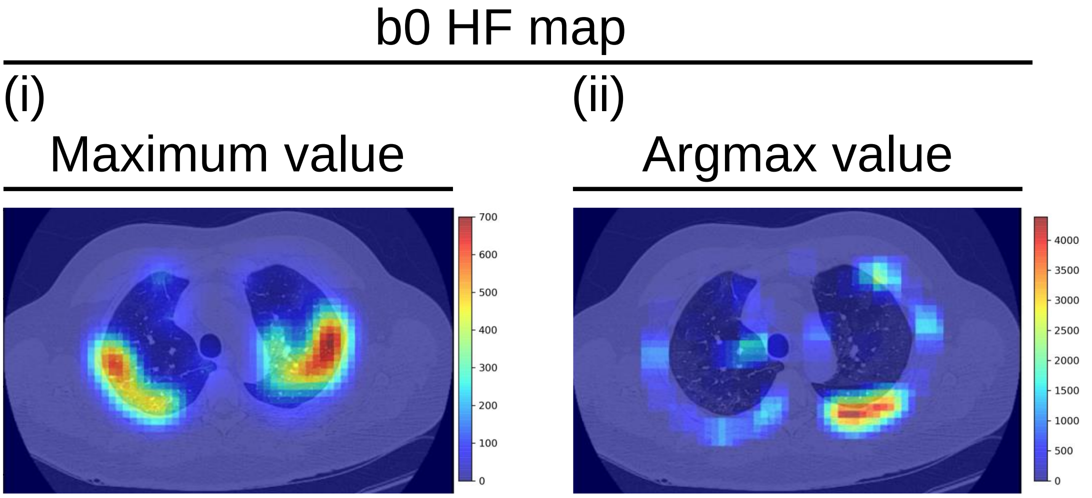

Online Resource 2. Preliminary validation of the effective HP-based statistical value on generating the HF map. (a) depicts an example CT image we used for the validation. Red circles indicate fibrotic lesions. (b) represents the results of the validation. (i) and (ii) were generated with the maximum value and the argmax value, respectively. According to this validation, it has been demonstrated that the b0 HF map generated with the maximum value of HP is more suitable for quantifying the fibrotic lesions in the CT image than with the argmax values.

Abbreviations: HP, homology-profile; HF, homology-based feature; and CT, computed tomography.
